# Genetic Polymorphisms Associated with Hearing Threshold Shift in Subjects during First Encounter with Occupational Impulse Noise

**DOI:** 10.1371/journal.pone.0130827

**Published:** 2015-06-29

**Authors:** Yohann Grondin, Magda E. Bortoni, Rosalinda Sepulveda, Elisa Ghelfi, Adam Bartos, Douglas Cotanche, Royce E. Clifford, Rick A. Rogers

**Affiliations:** 1 Molecular and Integrative Physiological Sciences Program, Department of Environmental Health, Harvard School of Public Health, 665 Huntington Ave, Boston, MA, 02115, United States of America; 2 Department of Otolaryngology-Head and Neck Surgery, 34800 Bob Wilson Dr., Suite 200, Naval Medical Center, San Diego, CA, 92134, United States of America; Harvard University, UNITED STATES

## Abstract

Noise-induced hearing loss (NIHL) is the most significant occupational health issue worldwide. We conducted a genome-wide association study to identify single-nucleotide polymorphisms (SNPs) associated with hearing threshold shift in young males undergoing their first encounter with occupational impulse noise. We report a significant association of SNP rs7598759 (p < 5 x 10^-7^; p = 0.01 after permutation and correction; Odds Ratio = 12.75) in the gene coding for nucleolin, a multifunctional phosphoprotein involved in the control of senescence and protection against apoptosis. Interestingly, nucleolin has been shown to mediate the anti-apoptotic effect of HSP70, a protein found to prevent ototoxicity and whose polymorphisms have been associated with susceptibility to NIHL. Increase in nucleolin expression has also been associated with the prevention of apoptosis in cells undergoing oxidative stress, a well-known metabolic sequela of noise exposure. To assess the potential role of nucleolin in hearing loss, we tested down-regulation of nucleolin in cochlear sensory cells HEI-OC1 under oxidative stress conditions and report increased sensitivity to cisplatin, a chemotherapeutic drug with ototoxic side effects. Additional SNPs were found with suggestive association (p < 5 x 10^-4^), of which 7 SNPs were located in genes previously reported to be related to NIHL and 43 of them were observed in 36 other genes previously not reported to be associated with NIHL. Taken together, our GWAS data and *in vitro* studies reported herein suggest that nucleolin is a potential candidate associated with NIHL in this population.

## Introduction

Noise exposure is a primary cause of hearing loss with a broad range of secondary non-auditory effects including cognitive impairment, sleep disturbance and cardiovascular disease [1]. Noise-induced hearing loss (NIHL), which correlates highly with tinnitus, is the most common and significant occupational health issue, affecting 16% of the population worldwide [2].

In the inner ear, noise exposure affects the lateral wall, organ of Corti and afferent neurons resulting in hearing impairment categorized as either temporary or permanent hearing threshold shifts. Although there are morphological and functional differences associated with either form of hearing impairment, the molecular basis for that difference is not well defined. However, it is well established that the permanence of hearing loss resides principally in the irreversible loss of cochlear hair cells in the organ of Corti [3, 4, 5], with some contribution of the stria vascularis [6] and afferent neurons [7]. Numerous reports suggest oxidative stress resulting from increased levels of reactive oxygen species after noise exposure is the predominant contributing mechanism to the loss of these hair cells [5, 8]. This increase in reactive oxygen species damages mitochondria, triggering the release of pro-apoptotic factors that activate a cellular apoptotic response [5, 8]. Loss of hair cells by apoptosis following noise exposure may also result from extracellular potassium dysregulation through alteration of cell-cell junctions between hair cells and Hensen's cells in the organ of Corti [3, 8, 9].

Supporting the role of oxidative stress and apoptosis in hearing loss, administration of antioxidants in animal models exposed to noise and inhibition of apoptotic signaling pathways, respectively, have shown efficacy in preventing cochlear hair cell loss [10–12] and is bio-available through several routes to the cochlea [13].

The susceptibility of individuals to NIHL results from both environmental factors that increase physiological stress, inflammation and oxidative stress [3, 6, 14]; and from genetic factors [15]. Genomic studies utilizing candidate gene analyses or genome-wide association studies (GWAS) have identified some of the genetic variants associated with NIHL susceptibility [15]. Coincidental with the known mechanisms of hearing loss, these studies have reported polymorphisms in oxidative stress response pathways, involving HSP70 and SOD1 proteins; as well as potassium recycling pathways, involving KCNQ4 and KCNE2 proteins [15].

Here, we report the results of a GWAS identifying a novel susceptibility locus associated with NIHL in 41 individuals selected amongst 314 age-controlled subjects during their first encounter with occupational noise. We then validate the potential role of nucleolin, the top candidate gene containing a significant SNP in our study, by developing a HEI-OC1 cochlear cell with silenced nucleolin, then experimentally testing its susceptibility to oxidative stress conditions and to ototoxic drug cisplatin.

## Materials and Methods

### Ethics Statement

The protocol for the collection of saliva samples and the identification of biomarkers of susceptibility to NIHL by GWAS was reviewed and approved by the Institutional Review Board at Naval Medical Center, San Diego. The use of de-identified saliva samples for DNA analysis was further reviewed by the Institutional Review Board at Harvard and received a non-human subject approval. All volunteers enrolled in the study provided written informed consent for saliva collection and its use for the identification of genetic markers. Samples were de-identified, anonymized and an identification number was randomly assigned to each subject. At the time of the saliva collection, samples were labeled with the randomized identification number used throughout the analysis.

### Subject Selection Criteria for Inclusion in the Marine Recruit Study

Subjects were randomly selected from a pool of volunteer participants enrolled in the placebo arm of the Marine Recruit Study “*Prevention of Noise-Induced Hearing Loss Using the Antioxidant Supplement*, *N-acetylcysteine*: *Impulse Noise Study* (*NMCSD*.*2007*.*0013*)”. Briefly, subjects were in good health as determined by enlistment criteria, without previous exposure to military noise. For admission into the study, subjects were required to have normal hearing in both ears (no hearing threshold greater than 25 dB HL (Hearing Level) at any of the 1, 2, 3, 4, 6 kHz standard audiometric test frequencies and no more than 30 dB SPL (Sound Pressure Level) asymmetry between ears at 8 kHz). Subjects were required to have normal tympanometry on baseline and on the day of final hearing testing, 5 weeks after pre-exposure audiogram. Recruits with abnormal tympanograms consistent with middle ear pathology and those with prior history of head trauma were excluded. The average age of the study participants was 19.82 years old.

### Noise Exposure

During their training period, subjects underwent 10 days of small arms training with an M16 rifle, which commonly generates an approximate 157 dB peak SPL impulse noise [16]. More precisely, noise exposure was spread over a period of 16 days in 2 series of 5 days with a period of 6 days in-between. The bulk of the firing occurred during the first 5 days of the 16 days period. Each subject fired up to 325 rounds of ammunition and was monitored for their use of hearing protective devices, consisting of foam ear-plugs in accordance with standard operating procedures. Standard military foam ear plugs used had a NRR of 29 dB (Noise Reduction Rating) with expected attenuation of 22 dBA as per OSHA standard.

### Audiogram Collection

Subjects underwent pre-exposure audiograms 6 to 7 days prior to noise exposure. A post-exposure audiogram was administered 12 days after last noise exposure which took place 36 to 37 days after the pre-noise exposure audiogram. At the time of the post-exposure audiograms, every threshold shift greater than 5 dB HL compare to pre-exposure audiogram was checked twice. Subjects with a hearing change also underwent a final tympanogram to rule out middle ear pathology as a cause of the hearing loss. Audiograms were performed by certified audiologists on clinical audiometer Interacoustics AC40 (Minneapolis, MN/USA) with a 5 dB step size sound level, and using TDH-39P earphones for 500 Hz and 1, 2, 3, 4, 6 kHz and Koss Pro/KTX-6 earphones (Milwaukee, WI/USA) for 8 kHz. Earphones were calibrated daily using OSCAR electro-acoustic ear simulators (Tremetrics, Eden Prairie, MN/USA) and data were collected in double-walled sound-attenuated booths (Acoustics Systems, Austin, TX/USA).

### Selection Criteria for GWAS Analysis

In the study reported here, only the Marine recruits from the control arm (placebo) of the retrospective impulse noise study (NMCSD.2007.0013) were included. Subjects were divided in hearing loss versus no hearing loss groups by calculating the mean threshold shift per subject. Briefly, the threshold shifts between post- and pre-exposure audiograms were first calculated for each ear at 2, 3 and 4 kHz frequencies respectively (n = 6 data points per individual) and then averaged for each subject. The mean threshold shift provides a unified single parameter that best captures all combinations by which hearing threshold may change, and is associated with conversational frequencies most relevant to future impact on the ability to hear the spoken word. Subjects were considered to have hearing loss if the mean threshold shift between pre- and post-exposure of both ears at frequencies 2, 3 and 4 kHz was greater than zero. In contrast, subjects had no hearing loss if the mean threshold shift of both ears at frequencies 2, 3 and 4 kHz was less or equal to 0. For this study, a total of 48 samples were included, consisting of 23 samples randomly selected from a pool of 204 subjects who sustained no hearing loss and 25 samples randomly selected from a pool of 110 subjects who sustained hearing loss. None of the selected samples presented more than 20 dB SPL asymmetry between ears at 8 kHz for either pre- or post-exposure audiograms.

### Genotyping and Quality Controls

The DNA isolated from the de-identified saliva samples collected at the time of the post-exposure audiogram was genotyped at Boston University Microarray Core Resource facility on Genome-Wide Human SNP Array 6.0 (Affymetrix, Santa Clara, CA).

Quality controls of the genotyped DNA were performed following GWAS standards [17]. Samples or SNPs failing these controls were removed from further analysis. From the genotype call using Birdsuite 1.5.5 [18], five samples were removed due to high variance estimate. For SNPs quality control, SNPs with a minor allele frequency < 5%, a genotyping call rate per SNP < 99%, or in Hardy-Weinberg disequilibrium (p < 10^−5^) were removed. Individuals with missingness > 1.5% were also removed. The genetic gender was checked with PLINK [19] and all matched to the gender reported in the phenotype file for all samples.

Samples were tested for duplicates and relatedness between subjects. For this a subset of autosomal SNPs in approximate linkage equilibrium were selected in PLINK to determine pairwise Identity-By-Descent (IBD). Autosomal SNPs with a genotyping call rate per SNP > 0.999, minor allele frequency > 5%, and a 100% call rate per sample were pruned to be on linkage equilibrium using the default settings of PLINK, resulting in a pruned set of 31,362 SNPs per sample to test for duplicates and relatedness. Pi-hat values showed there was no duplicate sample (all pi-hat < 0.9) and only one pair of individuals showed relatedness (pi-hat > 0.185). The individual with the lowest genotyping rate of the related pair was removed.

The population structure was assessed by principal component analysis using SNPRelate [20] in R version 3.0.2 [21]. For this, the founder population of Hapmap phase 3 release 2 (988 individuals) [22] and the NIHL study population were merged with PLINK. SNPs with A/T and C/G variants were removed to prevent allele swap mismatch and only SNPs common to all datasets were used. SNPs were flipped to match with sense of the strand. From the principal component analysis on the merged population, principal components 1 and 2 were used to identify and remove population outliers from the sample and adjust for population stratification to prevent bias. Only one sample fell outside the reference clusters and was subsequently removed.

As a final quality control measure, we used quantile-quantile plot and the genomic inflation score λ < 1.00. In total, 41 samples out of the 48 genotyped samples passed the quality controls.

### Reagents for In Vitro Studies

The cochlear hair cell line HEI-OC1 [23] was a generous gift of Dr. Kalinec (House Ear Institute, Los Angeles, CA, USA). High-glucose Dulbecco's Modified Essential Medium (DMEM, cat no. 11965–092) and fetal bovine serum (cat no. 26140–111) were purchased from Life Technologies. Cisplatin (cat no. 479306) and Mission shRNA containing lentiviral particles were purchased from Sigma-Aldrich. ATPlite one step assay (cat no. 6016731) was purchased from Perkin Elmer and CellTox Green Cytotoxicity Assay (cat no. G8741) from Promega.

### Cell Culture and Silencing of Nucleolin

For propagation, HEI-OC1 cells were cultured in high-glucose DMEM with 10% FBS at the permissive temperature of 33°C and 5% CO_2_. All experiments were performed on cells transferred to the non-permissive temperature of 39°C, 5% CO_2_.

To determine the effect of nucleolin down-regulation on HEI-OC1 cells, we constructed stable clones expressing shRNA targeted against nucleolin. For this, lentiviral particles containing either shRNA directed against nucleolin or a scramble shRNA for control were transfected at the permissive temperature of 33°C following a modified version of the protocol provided by the manufacturer. Briefly, on day 1, cells were seeded in 96 well plates at 5,000 cells / well in 110 μL of media containing 8 μg/mL of Hexadimethrine Bromide (Sigma) and the equivalent of 1 MOI (multiplicity of infection) of lentiviral particles. On day 2, the media containing the lentiviral particles was removed and replaced with fresh media. On day 3, the media was replaced with media containing 8 μg/mL of puromycin, which was then replaced every 3 days. At 90% confluence, cells were detached using trypsin EDTA (0.25%) solution with phenol red (Life Technologies, cat. no. 25200056) and cultured on 10 cm tissue culture Petri dishes for colony selection and expansion. Nucleolin silencing was confirmed by qPCR and Western Blot. A HEI-OC1 nucleolin-silenced clone, labeled sh-Ncl, with greater than 50% silencing, as confirmed by RTqPCR and Western Blot, was chosen for the viability and cytotoxicity experiments. Cells from the scramble transfection, labeled sh-Scr, were used as control.

### RTqPCR

To assess silencing of the nucleolin gene, RNA was extracted using the RNeasy minikit from Qiagen and the Qiacube for semi-automated column processing, utilizing Qiashredder (Qiagen) for cell lysis and on-column DNA digestion (RNase free DNase Set, Qiagen). Extracted RNA was quantified using Take-3 plate from Biotek and Biotek’s Synergy H1 multiplate reader. 40 ng of RNA were used for a 20 μL reverse transcription reaction using SuperScript III enzyme from Invitrogen, following the manufacturer’s protocol. The qPCR was done using the LC96 equipment and FastStart Universal SYBR Green PCR Master Mix from Roche. 10 μL reactions were done with 5 μL of a 1:10 cDNA dilution, following the recommendation of the manufacturer and an annealing temperature of 59°C. Primer pairs for nucleolin and reference gene β-actin were: Nucleolin Forward: 5’-CAGAACCCACATGGCAAACC-3’; Nucleolin Reverse: 5’-GCCTGATTGTTCTGCCCTCA-3’; β-actin Forward: 5’-ATGTGGATCAGCAAGCAGGA-3’; β-actin Reverse: 5’-AAGGGTGTAAAACGCAGCTC-3’.

### Western Blot

To assess silencing of nucleolin protein, cells at 80% confluence were lysed directly on the plate using NP-40 lysis buffer (10% Glycerol, 1% NP-40, 1mM MgCl2, 20 mM Tris-HCLpH4, 150 mM NaCl, and 50 mM B-glycerophosphate in water) supplemented with protease and phosphatase inhibitor cocktail (Thermo Scientific, Cat. No. 78420 and Sigma Aldrich, Cat. No. P8340, respectively) for 15 min on ice. Protein concentration was determined via Bradford assay, and 15 μg of protein were prepared in loading buffer composed of Laemmli (Biorad 161–0737) and 2-Mercaptoethanol solution (Sigma M3148) prepared according to the manufacturer’s instructions, boiled for 5 min and loaded into a 12% acrylamide gel. Electrophoresis and protein transfer using a PVDF membrane (Biorad 162–0177) was performed following the protocol provided by Abcam. Primary antibody (AB22758, Abcam) was diluted 1:1000 in blocking buffer and incubated overnight at 4°C and secondary antibody (AB7074, Cell Signaling) was diluted 1:5000 and incubated for 3 h. The blot was developed using Clarity Western ECL substrate (Bio-Rad 170–5060). Experiments were done in duplicate.

### Viability and Cytotoxicity

Nucleolin silenced clone sh-Ncl and scramble clone sh-Scr were seeded in 96-well plates at a density of 8,000 cells / well in 100 μL of media and cultured overnight under permissive temperature conditions for a confluence of 50% to 60%. Thereafter, the cells were transferred to non-permissive temperature conditions. After overnight incubation, cells reached 80% to 90% confluence and were treated with cisplatin (50 μL of 20 μM cisplatin in culture media) for 6h, in non-permissive conditions. Cell membrane damage and ATP production were multiplexed in 96-well plates using CellTox Green Cytotoxicity Assay and ATPlite one step assay, following the respective manufacturer’s instructions. The CellTox Green Cytotoxicity assay was performed first, followed by ATPlite assay. Briefly, a 1:500 dilution working stock of the CellTox Green dye was prepared in assay buffer and added in a 1:1 ratio to the wells at 100 μL per well. Plates were mixed at 700 rpm for 1 min in a microplate shaker and left to equilibrate at room temperature (RT) for 15 min. Fluorescence was measured using the Synergy H1 multiplate reader (Biotek), at an excitation/emission wavelengths of 485/528 nm respectively. 100 μL of volume were removed from each well and discarded. To perform the ATPlite one step assay, an equal volume of reconstituted reagent (100 μL) was added per well with plate mixed at 700 rpm for 2 min in a microplate shaker. Luminescence was then read using Synergy H1 multiplate reader. The assay was performed in duplicate with n = 4.

### Immunocytochemistry and Confocal Microscopy

To evaluate the altered expression of nucleolin protein in sh-Ncl clones, we undertook immunocytochemical staining of nucleolin in cells in culture. In normal cells, nucleolin is associated to the fibrillar components in nucleoli [24]. Immunocytochemistry labeling protocols were applied to cells seeded in Lab-Tek II 4-well chamber slides (Nalgen Nuc) [25]. After incubation, wells were washed from residual culture media with Dublecco’s Phosphate Buffered Saline (DPBS) and Tris-Buffered Saline solution with 0.1% Tween 20 (TBS-T), then fixed in 4% paraformaldehyde in PBS pH 7.2, freshly diluted from 16% paraformaldehyde, for 10 min at RT. Cells were briefly washed in TBS-T then incubated for 10 min in permeabilizing buffer (0.2% saponin in TBS-T) rinsed in TBS-T. To reduce non-specific staining, cells were incubated for 1 h at RT with blocking solution (2% normal goat serum, 0.3% bovine serum albumin, 0.3 M glycine in TBS-T, filter sterilized). Blocking solution was removed and primary antibody, mouse anti-Nucleolin in Rabbit (AB22758), in a 1:100 dilution in blocking solution was added and incubated overnight at 4°C. Wells were washed 3 times with TBS-T for 15 min each wash and incubated for 1 h with secondary antibody Goat Anti-Rabbit IgG with Texas Red (AB6719, Abcam) and Alexa Fluor 488 phalloidin (A12379, Life Technologies) in a 1:800 and 1:150 dilution, respectively, in blocking buffer. Cells were washed 3 times with PBS, 15 min each wash and mounted using antifade solution with DAPI (P-36931, Life Technologies). To assess antibody specificity, controls with secondary antibody but lacking primary antibody against nucleolin were used with HEI-OC1 transfected with scramble RNA (S1 Fig).

Images of immunostained cells were acquired on a LSM 700 (Carl Zeiss) confocal microscope with a 40x/1.3 objective lens configured to detect DAPI-labeled nuclei using the 405 nm laser, phalloidin-labeled filamentous actin cytoskeleton using the 488 nm laser, and the 555 nm laser for nucleolin. Quantitative analysis of the micrographs was performed to compare the abundance of nucleolin in the nucleus of the different cell types and in response to treatment. For this, single-stain controls were used to determine the background levels of nucleolin signal. In total, 15 to 22 cells for each cell type and treatment in multiple fields of view were acquired using identical settings. Intensity threshold was then used to distinguish nuclear regions of high nucleolin abundance versus more diffuse ones. The total intensity in nucleolin-abundant regions normalized by the total nuclear area was then calculated. All data acquisition and measurements were performed using Zen Black software (Carl Zeiss).

### Statistical Analysis

For GWAS, the association study using allelic frequency test based on case vs. control was performed with PLINK. Only the SNPs within 20 kb of a gene were considered as reported in the human genome build 18 (NCBI36). The genome-wide significance threshold was set to 5 x 10^−7^ and threshold for suggestive association was set to 5 x 10^−4^. Because of the small sample size, we also calculated empirical significance levels, corrected for multiple hypothesis, using 30,000 permutations for each SNP using PLINK [19]. These empirical p-values were used to confirm genome-wide significance. Linkage disequilibrium plot was generated with SNAP [26] using chromosomal position of SNPs given by 1000 genomes [27].

The range of statistical power of the study was estimated with the ‘twoStageGwasPower’ package in R [28], which is an implementation of the method for power calculation previously described [29]. One stage was considered for the calculation with NIHL prevalence at 16% worldwide [2] and with prevalence ranging from 21% [30] to over 50% [31] in military environment.

For *in vitro* studies of nucleolin-labeled cellular experiments, Student’s t-test was performed to assess the difference of viability and distribution of nuclear-bound nucleolin between sh-Scr HEI-OC1 and sh-Ncl HEI-OC1 clones. The difference was considered significant for p < 0.05.

## Results

**Fig 1:**
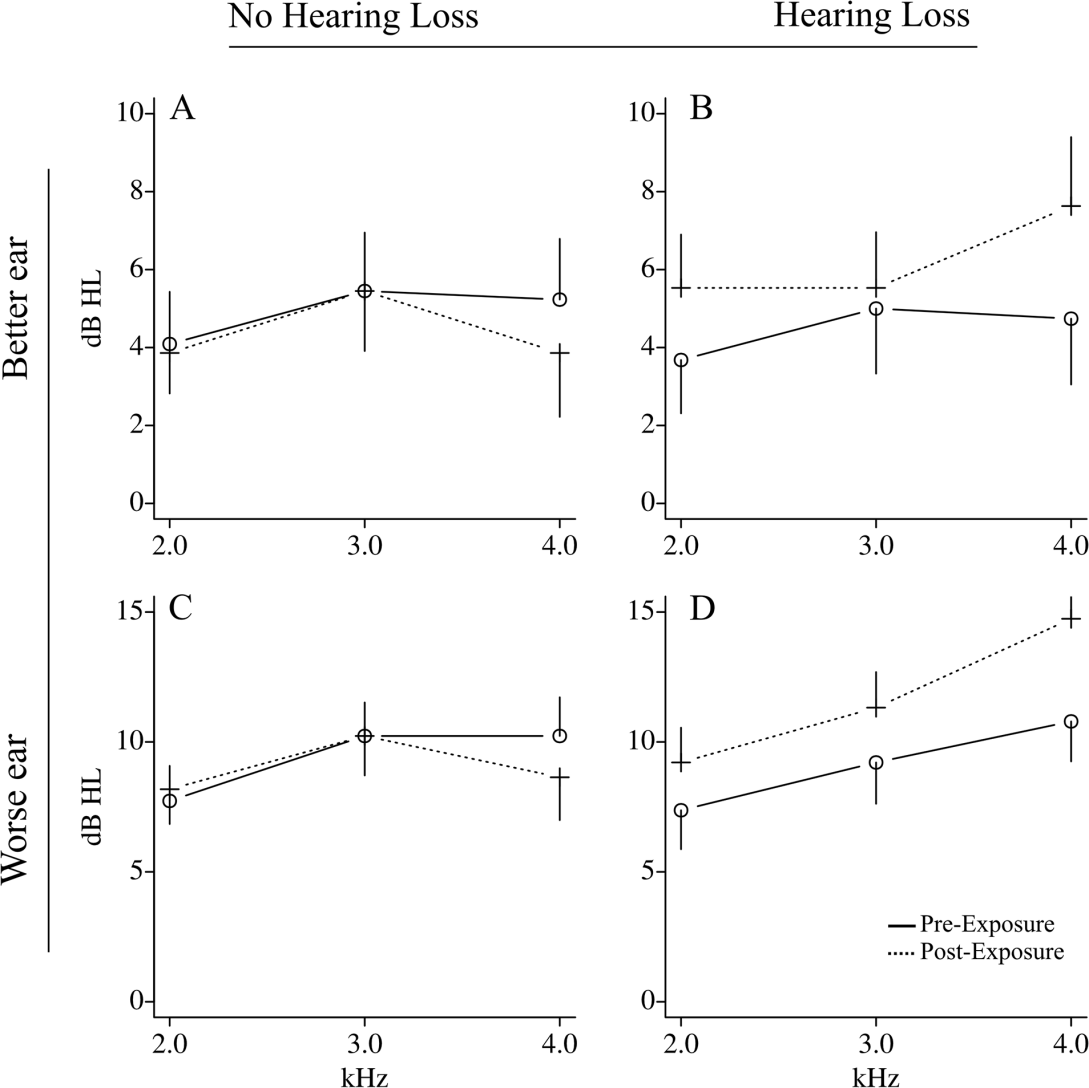
Composite audiograms of the hearing threshold pre- and post-noise exposure. Pre-noise exposure audiograms (circle, full line) and post-noise exposure audiograms (cross, dashed line) are shown for (A) the better ear of subjects without hearing loss; (B) the better ear of subjects with hearing loss; (C) the worse ear of subjects without hearing loss and (D) the worse ear of subjects with hearing loss. Data are the mean dB HL ± s.e.m at frequencies 2, 3 and 4 kHz.

We analyzed the association between genotype and hearing threshold shift of 41 subjects randomly selected from a pool of 314 young males exposed to repeat 157 dB SPL impulse noise for 10 days. Principal component analysis showed stratification in the sampled population with 25% of subjects (n = 10) overlapping with the Mexican ancestry cluster (MEX) and 75% (n = 31) overlapping with the European ancestry cluster (CEU) of the HapMap population (S2 Fig). After all quality controls and adjustment for population stratification, 289,036 genotyped SNPs and 41 genotyped samples of the 48 initially selected for the study remained for analysis. This consisted of 19 subjects experiencing hearing threshold shift and 22 subjects with no hearing loss. For the no hearing loss group, the mean threshold shift was -0.4 dB HL and for the hearing loss group, the mean threshold shift was 2.2 dB HL, showing a mean difference of 2.6 dB HL per frequency at 2, 3 and 4 kHz per ear between the two groups (p < 0.001, Student’s t-test). At the individual level, this mean threshold shift represents a difference of 15.6 dB HL per subject between the two groups. A unified representation of hearing changes using composite audiogram data shows that subjects within both groups responded differently to noise exposure (Fig 1). In the hearing loss group, subjects performed worse on the audiological test after noise exposure for either of the better or worse ear, consistent with a difference at conversational frequencies. In contrast, subjects experiencing no hearing loss performed similarly on their audiological test before and after noise exposure for either the better or worse ear.

### Genome-Wide Significant SNP Found in Nucleolin Gene

**Fig 2:**
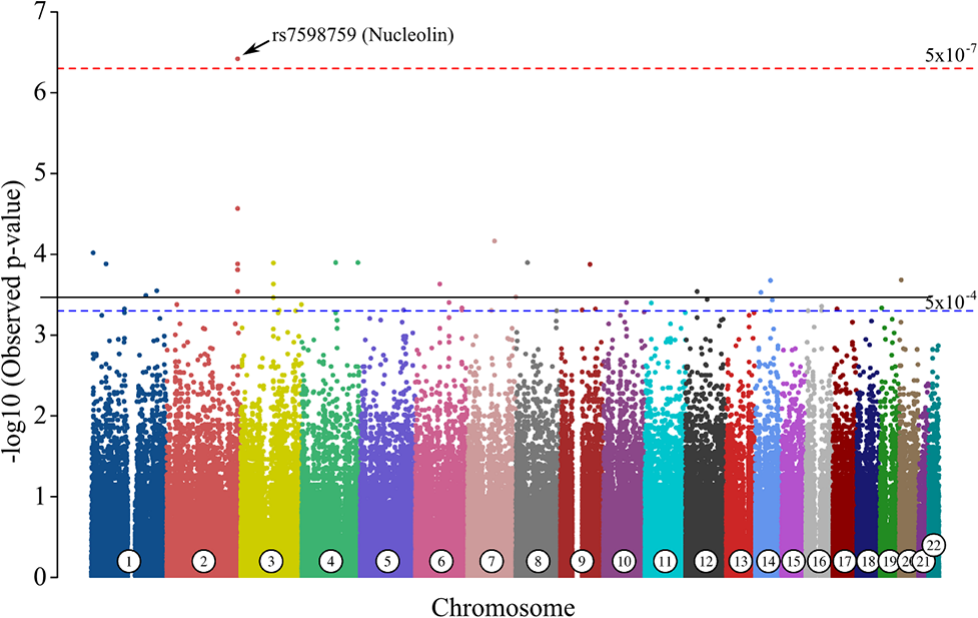
Manhattan plot of the SNPs associated with NIHL. Genome-wide significant SNP rs7598759 is located within nucleolin gene on chromosome 2 (black arrow). The-log10 of the p-value (y-axis) for each of the analyzed SNPs is shown with respect to their genomic location (x-axis) with chromosomes in alternate colors. Dashed lines indicate thresholds for genome-wide association (p < 5 x 10^−7^, red) and suggestive association (p < 5 x 10^−4^, blue).

**Fig 3:**
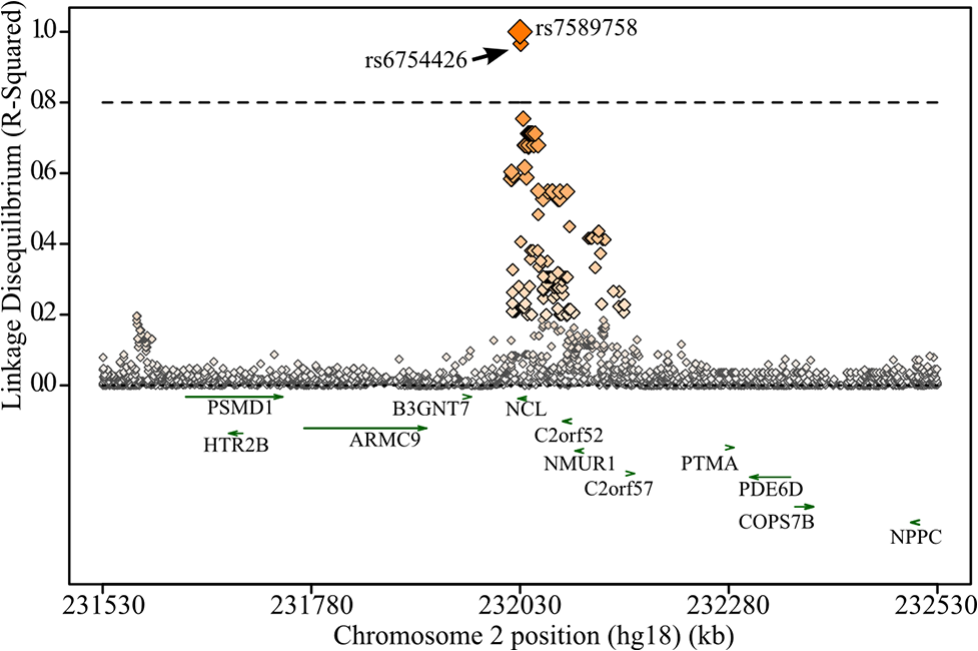
Regional linkage disequilibrium of newly identified SNP associated with NIHL. The location of genome-wide significant SNP, rs7598759 (top large filled diamond), is shown respective to the location of genes (green arrow and arrowhead, length based on size) within a 500 kb fragment of chromosome 2 (2q37.1). Genotyped SNP rs7598759 located within nucleolin gene (NCL) is in strong linkage disequilibrium (r^2^ > 0.9) with non-genotyped SNP rs6754426 (black arrow). Linkage disequilibrium (r^2^) is based on Hapmap CEU population with decreasing intensity of colored diamonds indicating decreasing linkage disequilibrium with SNP rs7598759. A SNP was considered in strong LD for r^2^ > 0.8 (black dashed line). The plot was generated with SNAP [26] using 1000 genomes [27] for the chromosomal position of SNPs.

We identified SNP rs7598759 associated at genome-wide significance (p < 5 x 10^−7^ and p = 0.01 after permutation and correction; Odds Ratio = 12.75) (Fig 2). This SNP is located in intron 9 of the nucleolin gene on chromosome 2q37.1 and contributes to a gain/loss of a CpG site. Additionally, two other SNPs, rs4973409 and rs4973410, located within the 20 kb flanking region of nucleolin, and one SNP, rs7571691, located in the gene *NMUR1* (neuromedin U receptor 1) contiguous to nucleolin were in suggestive association (p < 5 x 10^−4^). Linkage disequilibrium map showed genome-wide significant SNP rs7598759 is in strong linkage disequilibrium with the non-genotyped SNP rs6754426 (Fig 3) and the three SNPs in suggestive association (S1 Table).

### Additional SNPs of Interest

We identified 7 SNPs with suggestive evidence of association (p < 5 x 10^−4^) located in or within 20 kb of 7 genes with links to hearing loss (Table 1). We also identified another 43 SNPs with suggestive significance in or within 20 kb of 36 genes where no relation to hearing loss had been previously reported (Table 2). Of the 7 SNPs found in genes linked to hearing loss, three are of particular interest: two SNPs, rs7429015 and rs2436106, are found in genes coding for potassium channels which are important regulators of known pathways affected in hearing loss, and a third SNP, rs4374858, is found in gene encoding a membrane receptor protein. Of these, SNP rs7429015 is located in the intron of gene *KCNMB2* that encodes the potassium large conductance calcium-activated channel subunit β2; SNP rs2436106 is located within gene *KCNQ3* which is part of a family potassium voltage-gated channel protein associated with NIHL [32], [33]; and SNP rs4374858 is located within 20 kb of gene *GPR6*, which encodes a G protein-coupled receptor.

**Table 1:** Location of the SNPs with suggestive association with noise-induced hearing loss in genes previously related to hearing loss.

Chromosome	Gene	SNP^(a)^	Position^(b)^	Minor/Major Allele	P-value	OR
3	KCNMB2	rs7429015	179856818	A/G	5.00E-004	5.213
6	GPR6	rs4374858	110402134	G/C	3.95E-004	0.1901
8	KCNQ3	rs2436106	133191476	T/C	5.00E-004	5.213
8	RAB11FIP1	rs4956542	37877696	T/C	1.27E-004	22.36
10	PLAU	rs2227578	75348721	T/C	3.95E-004	0.1901
12	LGR5	rs10784926	70239349	C/T	3.61E-004	6.333
14	SLC39A9	rs7147710	68975478	T/C	2.11E-004	5.829

^**(a)**^SNPs are located in or within 20 kb of the listed gene.

^**(b)**^Position on chromosome based on NCBI build 36. OR–Odds ratio of the association.

**Table 2:** Location of the SNPs with suggestive association with noise-induced hearing loss in genes that have not been previously associated with hearing loss.

Chromosome	Gene	SNP^(a)^	Position^(b)^	Minor/Major Allele	P-value	OR
1	AGBL4	rs319981	48924473	G/A	1.32E-004	0.1605
1	CAMTA1	rs4908425	6823605	A/G	9.58E-005	9
1	FNDC7	rs11102337	109091734	C/G	4.74E-004	5.128
1	MR1	rs3845422	179279594	A/G	3.23E-004	0.1128
1	STXBP3	rs11102337	109091734	C/G	4.74E-004	5.128
2	C2orf52	rs7571691	232077645	C/T	1.32E-004	0.1605
2	C2orf52	rs6754952	232089489	T/G	2.89E-004	0.1773
2	LTBP1	rs4670387	33361950	T/A	4.18E-004	5.873
2	LTBP1	rs11124313	33366087	A/C	4.18E-004	5.873
2	NMUR1	rs7571691	232077645	C/T	1.32E-004	0.1605
2	NMUR1	rs1667305	232112073	A/G	1.56E-004	0.1667
2	NMUR1	rs1667313	232116262	A/C	1.56E-004	0.1667
2	NMUR1	rs6754952	232089489	T/G	2.89E-004	0.1773
3	NCBP2	rs746037	198160884	G/A	4.18E-004	5.873
3	PIGZ	rs746037	198160884	G/A	4.18E-004	5.873
3	SENP5	rs746037	198160884	G/A	4.18E-004	5.873
4	ELOVL6	rs7669237	111240190	G/A	1.27E-004	22.36
4	ELOVL6	rs10032613	111241207	A/T	1.27E-004	22.36
4	ODZ3	rs6839881	183966131	T/A	1.27E-004	22.36
5	ARHGAP26	rs4912893	142407999	T/C	4.89E-004	5.347
6	LCA5	rs9294147	80248251	C/T	2.33E-004	12.25
6	SYNE1	rs4645434	152706954	G/T	4.61E-004	5.559
6	SYNE1	rs9397102	152717547	T/C	4.61E-004	5.559
6	SYNE1	rs7747005	152705880	A/G	4.89E-004	5.347
7	MAGI2	rs11973067	78374514	C/T	4.95E-004	5.143
7	WDR60	rs6459913	158408025	G/A	3.38E-004	0.05225
9	PIP5K1B	rs1333340	70516547	G/C	4.89E-004	0.1846
9	PIP5K1B	rs4744685	70520218	G/A	4.89E-004	0.1846
9	PIP5K1B	rs2184117	70520904	T/G	4.89E-004	0.1846
9	SUSD1	rs10817281	113988347	G/C	4.74E-004	5.128
9	ZNF169	rs10993121	96063849	T/C	1.33E-004	0.1425
10	C10orf55	rs2227578	75348721	T/C	3.95E-004	0.1901
11	NELL1	rs4471417	21386046	G/C	4.00E-004	0.1515
12	CPNE8	rs10506125	37406554	C/T	2.89E-004	0.1773
14	CLEC14A	rs1028930	37781365	T/C	2.96E-004	7.02
14	ERH	rs897330	68906063	G/T	2.11E-004	5.829
14	GALNTL1	rs897330	68906063	G/T	2.11E-004	5.829
14	GALNTL1	rs12884348	68895853	T/C	5.00E-004	5.213
14	JDP2	rs3784013	75000823	G/T	3.67E-004	8.913
16	A2BP1	rs7196635	7693602	T/C	5.00E-004	5.213
16	FTO	rs7205213	52434067	T/C	4.40E-004	5.167
16	FTO	rs8053367	52432985	A/C	4.95E-004	5.143
16	FTO	rs8053740	52433213	G/C	4.95E-004	5.143
16	FTO	rs7203051	52433650	G/C	4.95E-004	5.143
16	FTO	rs7205009	52433945	T/C	4.95E-004	5.143
17	COX10	rs2159132	13946164	C/T	4.74E-004	5.128
19	FUT3	rs778809	5781302	A/G	4.61E-004	5.559
19	FUT3	rs17271883	5785212	A/G	4.61E-004	5.559
19	FUT6	rs778809	5781302	A/G	4.61E-004	5.559
19	FUT6	rs17271883	5785212	A/G	4.61E-004	5.559
19	NRTN	rs778809	5781302	A/G	4.61E-004	5.559
19	NRTN	rs17271883	5785212	A/G	4.61E-004	5.559
20	CRLS1	rs6053819	5930604	G/A	2.08E-004	5.963
20	MCM8	rs6053819	5930604	G/A	2.08E-004	5.963

^**(a)**^SNPs are located in or within 20 kb of the listed gene.

^**(b)**^Position on chromosome based on NCBI build 36. OR–Odds ratio of the association.

### Nucleolin Down-Regulation Increases Susceptibility to Oxidative Stress in Cochlear Cell Model

The present GWAS results reveal significant association of SNP rs7598759 within the nucleolin gene that encodes a multifunctional protein shown to be expressed in numerous cell types, including primary cochlear cell cultures of rats [34]. Given that nucleolin also interacts with proteins associated with various forms of hearing loss, including NIHL, its dysregulation in NIHL susceptibility is plausible. We therefore tested the effect of nucleolin on the viability of cochlear cells *in vitro*.

To test the potential effect of nucleolin dysregulation on hearing-loss, we utilized HEI-OC1 cochlear cell line commonly used as a model for drug-induced ototoxicity [35], [36]. These cells contain a temperature-sensitive mutant of SV40 large-T antigen allowing the cells to grow at permissive temperature (33°C) and differentiate at non-permissive temperature (39°C). Under cisplatin exposure, a chemotherapeutic drug responsible of hearing loss in 60% of pediatric patients [37], HEI-OC1 cells exhibit alterations of biomolecular pathways also known to be affected in NIHL [35], [36] and can be used as a surrogate for *in vitro* NIHL studies.

**Fig 4:**
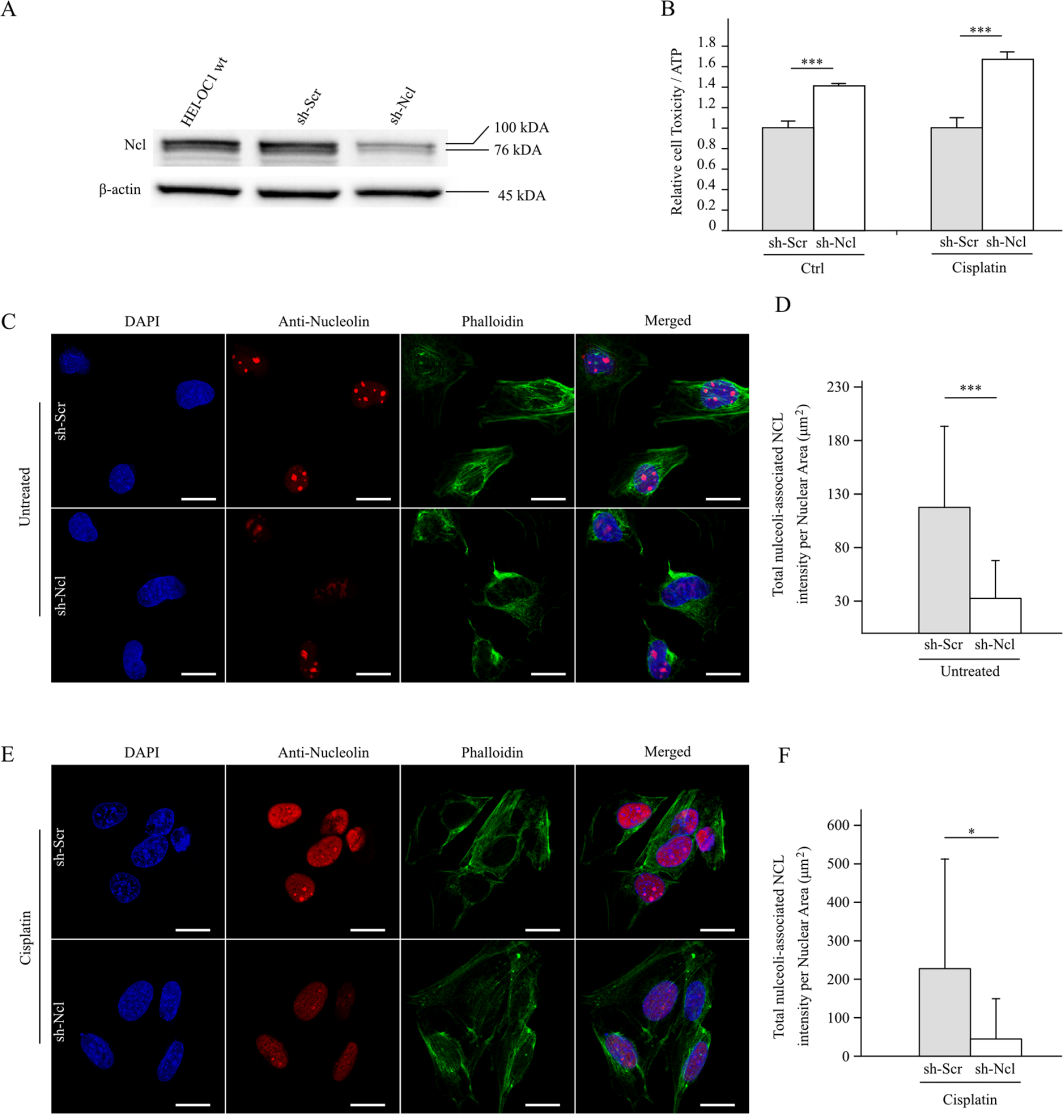
Down-regulation of nucleolin affects viability and nuclear redistribution in HEI-OC1 cells. (A) Western blots of two independent experimental replicates show HEI-OC1 wild type cells and transfected HEI-OC1 cells with either shRNA targeted against nucleolin (sh-Ncl) or with scramble shRNA control (sh-Scr). (B) Cytotoxicity of sh-Scr and sh-Ncl cells either untreateds or incubated 6 h with Cisplatin at 20 μM from duplicate experiments with n = 4. (C, D) Redistribution of nucleolin in shRNA-transformed HEI-OC1 cells. Transfected HEI-OC1 cells with shRNA targeted against nucleolin (sh-Ncl) or with scramble shRNA as control (sh-Scr) were untreated or (E, F) incubated with 20 μM Cisplatin for 6 h. Cells were stained with anti-nucleolin antibody decorated with secondary goat anti-rabbit IgG Texas-Red conjugate and counterstained with DAPIand phalloidin. Images were then merged to form color composite images. The images are representative of the results of each cell type and treatment. White scale bar represents 20 μm. The change in the total nucleolin intensity was normalized to nuclear area in untreated cells (D) or in cells incubated 6 h with cisplatin at 20μM (F). Statistical significance was assessed with Student's t-test (***p < 0.001, *p < 0.05). Experiments were performed in non-permissive conditions.

We constructed stable clones expressing shRNA targeted against nucleolin (sh-Ncl). In comparison to shRNA scramble control clones (sh-Scr), nucleolin expression in silenced sh-Ncl clones was significantly reduced (Fig 4A). The down-regulation of nucleolin in sh-Ncl clones significantly increased cellular toxicity in either untreated cells (p < 0.001, Student’s t-test) or under cisplatin-induced stress (p < 0.001, Student’s t-test) when compared to control cells (Fig 4B). This increase in sensitivity in cells expressing lower nucleolin was also reflected on the nuclear distribution of nucleolin as revealed by confocal microscopy. Expression of nucleolin in sh-Scr control clones was associated with the fibrillar components of the nucleus (Fig 4C), similar to previous reports in normal cells [38]. Down-regulation of nucleolin in sh-Ncl clones reduced the number of nucleoli-associated nucleolin (p < 0.001, Student’s t-test) (Fig 4D). Nuclear redistribution of nucleolin was also altered in sh-Scr control clones exposed to 20 μM of cisplatin for 6 h (Fig 4E) similar to that previously reported in the nucleus of mouse fibroblasts [39]. This redistribution was further altered in sh-Ncl clones with a further significant loss of nucleoli-associated nucleolin (p < 0.05, Student's t-test) (Fig 4F).

## Discussion

Previous susceptibility studies have identified NIHL associated genes mainly in pathways that regulate oxidative stress response and potassium channels. Our results show that novel candidate genes in these pathways are associated with NIHL. In particular, the results reported here suggest that nucleolin, a multifunctional phosphoprotein involved in the modulation of cell proliferation and protection against apoptosis [40, 41], is a strong candidate for NIHL susceptibility. This is supported by the numerous interactions nucleolin has with hearing loss-associated proteins, such as heat shock protein HSP70 [42, 43], pro-apoptotic protein P53 [44, 45] and mitochondrial-associated apoptosis protein Bcl2 [46]. The nature of the associations of these proteins with NIHL is varied and intervenes at several levels of regulation of gene expression and protein activity. For example, polymorphisms in the gene coding for HSP70 alone or in combination with genes coding for other heat shock proteins, are associated with NIHL susceptibility [14, 47] and sudden sensorineural hearing loss [48]. It has also been shown experimentally that under various cell stresses, nucleolin regulates P53 [45, 49], a pro-apoptotic protein that is found activated in outer hair cells and Hensen's cells shortly after impulse noise exposure [12]. Finally, over-expression of Hsp70 [50] and Bcl2 [51, 52] or inhibition of P53 [52] in rodents reduces aminoglycoside-induced ototoxicity.

All of these NIHL associated proteins, together with nucleolin, contribute to the apoptotic cellular response induced by oxidative stress and DNA damage, which are known to result in permanent hearing loss [5, 8]. The prevention of apoptosis in numerous human-derived cell lines under various stress conditions follows from an increased expression of nucleolin [53, 54] and appears to be mediated by interactions with NIHL-associated proteins Bcl2, HSPA1A (Heat Shock 70 Protein 1A) and p53. These interactions are essential in the regulation of these proteins, in particular under oxidative stress. For instance, results indicate that nucleolin exerts a regulatory control of HSPA1A [43] and that either its over-expression or increased stability by over-expression of HSPA1A in H_2_O_2_-exposed rat cardiomyocytes prevents apoptosis [42, 55]. It has also been shown that the interaction of nucleolin with RPL26 (ribosomal protein L26), a necessary protein for induction of P53 translation following DNA damage [45], prevents the translation of P53 and P53-dependent apoptosis [45, 49].

In the HEI-OC1 cochlear cell model, our *in vitro* results show that down-regulation of nucleolin decreased cell viability and support the role of nucleolin in the regulation of cell death of cochlear sensory cells. The effect of nucleolin down-regulation is actually consistent with reports in other cell models. Taken together, our GWAS data and *in vitro* studies reported herein suggest that nucleolin might be a potential target for therapeutic intervention in context with NIHL. No previous study has investigated the role of nucleolin in hearing loss, although other studies have shown that nucleolin dysregulation, together with the proteins it interacts with, Bcl2, P53 and HSP70, have been associated in a wide range of cancers, viral infections and autoimmune diseases [41].

The potassium recycling pathways that maintain the electrochemical gradient across cell compartments and mediate potassium currents in the cochlea are also essential for hearing. Consequently, mutations and polymorphisms in several genes involved in these pathways lead to or have been associated with hearing loss [15, 56]. These include potassium-gated voltage channel genes *KCNE1*, *KCNQ1* and *KCNQ4* [32, 33], potassium large-conductance calcium-activated channel subunit *KCNMA1* [57] and potassium inwardly-rectifying channel *KCNJ10* [33].

In this study, we identified two additional candidate potassium channels genes *KCNQ3* and *KCNMB2* associated with hearing threshold shift. Although *KCNQ3* has not been previously reported in association with hearing loss, family members of this gene, including *KCNQ1* and *KCNQ4*, have been reported associated with NIHL [32], [33] which supports the possible role of *KCNQ3*. Changes in *KCNQ3* activation alone have been linked with induction of tinnitus [58], while the dimerization of KCNQ3 with other potassium channels seems to be essential for hearing. For example, dimers of KCNQ3/4 modulate the dynamics of activation of hearing loss-associated candidate *KCNQ4* [59] and might contribute to the critical low-voltage activated potassium conductance in both inner and outer hair cells of mice [60]. In addition, another dimer, KCNQ2/3, has been shown to regulate apoptosis in cortical and hippocampal neurons [61].

Another candidate gene, *KCNMB2*, is a subunit of large-conductance calcium-activated potassium channels previously reported in linkage disequilibrium with rs4603971, another SNP in suggestive association with hearing loss [62]. In addition, KCNMB2 subunit interacts with KCNMA1 [63], the α-subunit of potassium large conductance calcium-activated channel which also presents NIHL-associated polymorphisms [57] and whose deletion produces progressive deafness [63]. Furthermore, both protein subunits are under regulation of interferon-γ [64], an inflammatory marker up regulated in cases of sensorineural hearing loss [65]. Our results independently confirm the potential role of *KCNMB2* for hearing loss susceptibility. Of interest, both candidate genes *KCNQ3* and *KCNMB2* as well as *KCNJ10* previously mentioned, appear to be associated with epilepsy [59, 66, 67], although only *KCNJ10* has been thus far associated with both epilepsy and hearing loss [33].

The third SNP, rs4374858, is located within 20 kb of gene *GPR6* and encodes a G protein-coupled receptor. Interestingly, this receptor shows a high affinity for sphingosine-1-phosphate [68], a sphingolipid metabolite that protects the cochlea against ototoxicity induced by gentamicin, an antibiotic member of aminoglycoside family [69].

Many of the SNPs identified in this study, including genome-wide significant SNP rs7598759 in nucleolin, are located within introns. The means by which these polymorphisms might contribute to altering the pathways in which the associated proteins are involved is unknown, although we can speculate that they could affect gene transcript levels, as previously reported [70]. This might occur through various regulatory mechanisms involving different classes of non-coding RNA, for example [71]. Considering also that the nucleolin SNP identified in this report contributes to a Gain/Loss of a CpG site, one such possible mechanism may involve changes in DNA methylation and epigenetic controls. Such changes may be under the constraint of environmental factors, which are actually known to significantly affect DNA methylation [72, 73] and we would anticipate noise exposure to be another form of environmental stress. Epigenetic control from environmental noise might be a strong component of the cochlear cell response and therefore sites with SNPs variants that alter DNA methylation might play a role in susceptibility to noise.

Does nucleolin dysregulation confer a predictive outcome for permanent threshold shift in humans? In practice, recovery from temporary threshold shift in human subjects varies from minutes to days. For example, human ears show significant recovery within hours from a 4-hour noise exposure at 100 dBA and full recovery within one week [74]. In more extreme cases in animals with severe noise exposures that generate up to 40 dB threshold shift at 24h, full recovery is observed within 2 weeks [75]. Based on the kinetics of recovery, we assume that the hearing loss experienced by some subjects at the post-exposure audiogram collected for the study would be permanent, which falls within the range of reported hearing loss for this population.

Many of the SNPs identified in this study are in genes that can potentially modulate apoptotic pathways and contribute to either temporary or permanent hearing loss. Indeed, although the loss of hair cells following activation of apoptotic signals clearly contributes to permanent hearing loss, it is not yet clear whether transient activation of these same pathways will contribute to temporary hearing loss [12]. Until further studies clarify this point, we suggest the apoptosis-related genes reported in this study contribute to either temporary or permanent NIHL.

In the placebo arm of the overall Marine recruit study, 35% of subjects experienced a threshold shift when exposed to occupational noise, which is well above the anticipated 16% from noise exposure studies conducted in other occupational settings worldwide [2]. Looking beyond the NIHL susceptibility polymorphisms identified in our study, we speculate that the environment training regimen undertaken by the subjects contributed to increased hearing loss and was likely mediated by physiological stress, inflammation, and oxidative stress [3, 6, 14].

We posit that strong environmental conditions, leading to exercise-induced inflammation and fatigue, contribute to NIHL susceptibility under certain occupational conditions. In this context, the state of inflammation and fatigue of an individual becomes a relevant risk factor of noise exposure in the work environment. Physiologic measures of stress, inflammation and oxidative stress were not evaluated in this study. However they are important factors to be explored in further studies to help place noise exposure in context with the genetic susceptibility to NIHL.

Intensive exercise or prolonged sleep restriction periods similar to Marine recruits training might result in altered hearing threshold shifts. Several reports have shown a significant increase in expression of inflammation markers C-reactive and IL-6 proteins [76, 77] under less extreme conditions than experienced by these Marine recruits. Reduction of exercise performance and fatigue resistance also correlates with an increase in IL-6 expression when ATP-activated protein kinase (AMPK) that senses cellular energy homeostasis to maintain ATP levels is altered [78]. It is therefore possible that the subjects in the study presented systemic levels of inflammation above baseline.

There is also growing evidence that hearing loss is a risk factor in inflammation-driven diseases such as diabetes [79] and dementia [80, 81, 82, 83] and that inflammation itself contributes to hearing loss. Data show that expression of inflammatory cytokines IL-1b and IL-6 in the inner ear increases shortly after noise exposure [84, 85] and that long-term inflammation measured by C-reactive protein correlates with hearing loss [86]. In addition, other reports suggest that inflammation levels correlate with the capability to recover from hearing loss injuries [87] and that blocking IL-6 signaling to suppress inner ear inflammation reduces NIHL [85].

To address the question of whether different sets of genes will have an effect on the susceptibility to either impulse or continuous noise, we compared the SNPs genotyped in our study to previously reported SNPs associated with NIHL. Of the 46 SNPs within 23 candidate genes previously reported associated with NIHL (Table 3), 15 SNPs were genotyped in our dataset. Of these 15 SNPs, the most significant SNP was found in gene *WSF1* (p < 7 x 10^−4^) identified in another impulse noise study [88]. In contrast, SNPs identified in continuous noise studies, such as in catalase and ITGA8 genes did not show association (p > 0.7). This result may suggest that different sets of candidate polymorphisms are specifically associated with either impulse or continuous noise exposure leading to hearing loss. Although impulse noise was the main component of this study of noise exposure, we cannot fully rule out the possibility of continuous noise exposure during the course of the study.

**Table 3:** List of published candidate genes with SNPs associated with NIHL.

Gene Symbol	Gene Name	References
CAT	Catalase	[89]
GJB1	Gap junction, beta 1	[33]
GJB2	Gap junction, beta 2	[33]
GJB4	Gap junction, beta 4	[33]
GRHL2	Grainyhead-like 2	[33]
GSTM1	Glutathione S-transferase mu 1	[90], [91]
GSTP1	Glutathione S-transferase pi 1	[90]
GSTT1	Glutathione S-transferase theta 1	[90], [91]
HSPA1A	Heat shock 70kDa protein 1A	[47], [92], [93]
HSPA1B	Heat shock 70kDa protein 1A	[47], [92], [93]
HSPA1L	Heat shock 70kDa protein 1-like	[47], [92], [93]
ITGA8	Integrin alpha 8	[33]
KCNE1	Potassium voltage-gated channel, member 1	[32], [33]
KCNJ10	Potassium inwardly-rectifying channel, subfamily J, member 10	[33]
KCNMA1	Potassium large conductance calcium-activated channel, alpha member 1	[33]
KCNQ1	Potassium voltage-gated channel, KQT-like subfamily, member 1	[32], [33]
KCNQ4	Potassium voltage-gated channel, KQT-like subfamily, member 4	[32], [33]
MYH14	Myosin, heavy chain 14, non-muscle	[33]
PCDH15	Protocadherin 15	[33]
PON2	Paraoxonase 2	[94]
POU4F3	POU domain, class 4, transcription factor 3	[33]
SOD2	Superoxide dismutase 2	[94], [95]
WFS1	Wolfram syndrome 1	[88]

Although the small sample size is a limiting factor, many of the results shown here converge with previously reported data and add more weight to previously identified polymorphisms associated with NIHL. Estimation of the power of this study, which depends on the NIHL prevalence and genetic model selected, is highly variable (0.02 < *β* < 0.63), challenging its objective interpretation. However, the results of *in vitro* experiments confirm the main finding of this study supporting the potential role of nucleolin in NIHL susceptibility.

## Conclusion

We conducted the discovery phase of a GWAS in subjects undergoing first encounter with occupational impulse noise and identified nucleolin as a new candidate gene associated with NIHL susceptibility. This study is the first to suggest the role of nucleolin in NIHL. We have further identified 7 genes with suggestive association that are known to be involved in hearing-loss pathways and 36 additional genes that have not previously been directly related to hearing-loss pathways. Further studies are needed to confirm the relevance of these polymorphisms to NIHL. Nucleolin was tested in a cochlear cell model and reveals susceptibility to ototoxic stress. Taken together, our GWAS data and *in vitro* studies reported herein suggest that nucleolin is a potential candidate associated with NIHL in this population.

## Supporting Information

S1 Fig Antisera specificity control for nucleolin antibody decoration in HEI-OC1 cells. Cultured HEI-OC1 cells with scramble shRNA control immuno-decorated with (B) goat anti-rabbit IgG Texas-Red conjugate used as antibody specific control. Cells were further counterstained with (A) DAPI to show nuclear profile and (C) phalloidin Alexa 488 conjugate to show actin cytoskeletal features. (D) The merge panel clearly shows nuclear and actin profiles and absence of non-specific staining. White scale bar represents 20 μm.
(TIF)

S2 Fig Principal component analysis of the sampled population. This figure demonstrates the results of the principal component analysis to identify stratification in order to avoid bias. Eleven sub-populations from 1000 Genomes were tested to stratify the NIHL study population. Principal component 1, defined as axis of the linear least square fit of the tested population and principal component 2, which is defined as second axis of the linear least square fit orthogonal to principal component 1, show the largest genetic variance between ASW (African ancestry in Southwest USA), CEU (Utah residents with Northern and Western European ancestry), CHB (Han Chinese in Beijing, China), CHD (Chinese in Metropolitan Denver), GIH (Gujarati Indians in Houston), JPT (Japanese in Tokyo), LWK (Luhya in Webuye, Kenya), MEX (Mexican ancestry in Los Angeles), MKK (Maasai in Kinyawa, Kenya), TSI (Toscans in Italy), YRI (Yoruba in Ibadan).
(TIF)

S1 Table Linkage disequilibrium between genome-wide SNP and suggestive SNPs in the nucleolin locus. Genome-wide significant (peak) SNP rs7598759 is in strong linkage disequilibrium (LD) with the three suggestive SNPs (D' > 0.9) although some the correlation are low due to lower allele frequency (r^2^ < 0.8). Linkage disequilibrium and distances between pairs of SNPs were calculated from either the 1000 Genomes [27] or HapMap release 22 [22] based on the CEU population.
(XLSX)

## References

[1] Basner M, Babisch W, Davis A, Brink M, Clark C, Janssen S, et al (2014) Auditory and non-auditory effects of noise on health. Lancet 383: 1325–1332. 10.1016/S0140-6736(13)61613-X 24183105 PMC3988259

[2] Nelson DI, Nelson RY, Concha-Barrientos M, Fingerhut M (2005) The global burden of occupational noise-induced hearing loss. Am J Indor:park Med 48: 446–458. 10.1002/ajim.20223 16299704

[3] Ohlemiller KK (2008) Recent findings and emerging questions in cochlear noise injury. Hear Res 245: 5–17. 10.1016/j.heares.2008.08.007 18790034 PMC2610263

[4] Op de Beeck K, Schacht J, Van Camp G (2011) Apoptosis in acquired and genetic hearing impairment: the programmed death of the hair cell. Hear Res 281: 18–27. 10.1016/j.heares.2011.07.002 21782914 PMC3341727

[5] Böttger EC, Schacht J (2013) The mitochondrion: a perpetrator of acquired hearing loss. Hear Res 303: 12–19. 10.1016/j.heares.2013.01.006 23361190 PMC3681877

[6] Shi X (2011) Physiopathology of the cochlear microcirculation. Hear Res 282: 10–24. 10.1016/j.heares.2011.08.006 21875658 PMC3608480

[7] Moser T, Predoehl F, Starr A (2013) Review of hair cell synapse defects in sensorineural hearing impairment. Otol Neurotol 34: 995–1004. 10.1097/MAO.0b013e3182814d4a 23628789

[8] Henderson D, Bielefeld EC, Harris KC, Hu BH (2006) The role of oxidative stress in noise-induced hearing loss. Ear Hear 27: 1–19. 16446561 10.1097/01.aud.0000191942.36672.f3

[9] Zheng G, Hu BH (2012) Cell-cell junctions: a target of acoustic overstimulation in the sensory epithelium of the cochlea. BMC Neurosci 13: 71 10.1186/1471-2202-13-71 22712683 PMC3407512

[10] Fetoni AR, De Bartolo P, Eramo SLM, Rolesi R, Paciello F, Bergamini C, et al (2013) Noise-induced hearing loss (NIHL) as a target of oxidative stress-mediated damage: cochlear and cortical responses after an increase in antioxidant defense. J Neurosci 33: 4011–4023. 10.1523/JNEUROSCI.2282-12.2013 23447610 PMC6619303

[11] Lu J, Li W, Du X, Ewert DL, West MB, Stewart C, et al (2014) Antioxidants Reduce Cellular and Functional Changes Induced by Intense Noise in the Inner Ear and Cochlear Nucleus. J Assoc Res Otolaryngol. 15: 353–372 10.1007/s10162-014-0441-4 24497307 PMC4010594

[12] Fetoni AR, Bielefeld EC, Nicotera T, Henderson D (2014) A putative role of p53 pathway against impulse noise induced damage as demonstrated by protection with pifithrin-alpha and a Src inhibitor. Neurosci Res: 1–8. 10.1016/j.neures.2014.01.006 24472721

[13] Grondin Y, Cotanche DA, Manneberg O, Molina R, Treviño-villarreal JH, Sepulveda R, et al (2013) Pulmonary delivery of D-methionine is associated with an increase in ALCAR and glutathione in cochlear fluids. Hear Res 298: 93–103. 10.1016/j.heares.2012.12.011 23296212

[14] Meltser I, Tahera Y, Canlon B (2010) Differential activation of mitogen-activated protein kinases and brain-derived neurotrophic factor after temporary or permanent damage to a sensory system. Neuroscience 165: 1439–1446. 10.1016/j.neuroscience.2009.11.025 19925854

[15] Sliwinska-Kowalska M, Pawelczyk M (2013) Contribution of genetic factors to noise-induced hearing loss: a human studies review. Mutat Res 752: 61–65. 10.1016/j.mrrev.2012.11.001 23207014

[16] US Army Center for Health Promotion and Preventive Medicine (2008) Noise levels of common army equipment.

[17] Anderson CA, Pettersson FH, Clarke GM, Cardon LR, Morris AP, Zondervan K (2010) Data quality control in genetic case-control association studies. Nat Protoc 5: 1564–1573. 10.1038/nprot.2010.116 21085122 PMC3025522

[18] Korn JM, Kuruvilla FG, McCarroll SA, Wysoker A, Nemesh J, Cawley S, et al (2008) Integrated genotype calling and association analysis of SNPs, common copy number polymorphisms and rare CNVs. Nat Genet 40: 1253–1260. 10.1038/ng.237 18776909 PMC2756534

[19] Purcell S, Neale B, Todd-Brown K, Thomas L, Ferreira MAR, Bender D, et al (2007) PLINK: a tool set for whole-genome association and population-based linkage analyses. Am J Hum Genet 81: 559–575. 17701901 10.1086/519795 PMC1950838

[20] Zheng X, Levine D, Shen J, Gogarten SM, Laurie C, Weir B, et al (2012) A High-performance Computing Toolset for Relatedness and Principal Component Analysis of SNP Data Title. Bioinformatics 28: 3326–3328. 10.1093/bioinformatics/bts606 23060615 PMC3519454

[21] R Core Team (2013) R: A Language and Environment for Statistical Computing. R Foundation for Statistical Computing, Vienna, Austria.

[22] The International Hapmap Consortium (2005) A haplotype map of the human genome. Nature 437: 1299–1320 16255080 10.1038/nature04226 PMC1880871

[23] Kalinec GM, Webster P, Lim DJ, Kalinec F (2003) A cochlear cell line as an in vitro system for drug ototoxicity screening. Audiol Neurootol 8: 177–189. 12811000 10.1159/000071059

[24] Ginisty H, Sicard H, Roger B, Bouvet P (1999) Structure and functions of nucleolin. J Cell Sci 112 6: 761–772. 10036227 10.1242/jcs.112.6.761

[25] Treviño-Villarreal JH, Cotanche D, Sepúlveda R, Bortoni ME, Manneberg O, Udagawa T, et al (2011) Host-derived pericytes and Sca-1+ cells predominate in the MART-1- stroma fraction of experimentally induced melanoma. J Histochem Cytochem 59: 1060–1075. 10.1369/0022155411428078 22147606 PMC3283083

[26] Johnson AD, Handsaker RE, Pulit SL, Nizzari MM, O’Donnell CJ, de Bakker P (2008) SNAP: a web-based tool for identification and annotation of proxy SNPs using HapMap. Bioinformatics 24: 2938–2939. 10.1093/bioinformatics/btn564 18974171 PMC2720775

[27] The 1000 Genomes Project Consortium (2012) An integrated map of genetic variation from 1,092 human genomes. Nature 491: 56–65. 10.1038/nature11632 23128226 PMC3498066

[28] Moore DF (2012) twoStageGwasPower: Compute thresholds and power for two-stage gwas.

[29] Skol AD, Scott LJ, Abecasis R, Boehnke M (2006) Joint analysis is more efficient than replication-based analysis for two-stage genome-wide association studies. Nature Genetics 38: 209–213. 16415888 10.1038/ng1706

[30] Yankaskas K (2013) Prelude: Noise-induced tinnitus and hearing loss in the military. Hear Res 295: 3–8. 10.1016/j.heares.2012.04.016 22575206

[31] Collee A, Legrand C, Govaerts B, Veken P, De Boodt F, Degrave E (2011) Occupational exposure to noise and the prevalence of hearing loss in a Belgian military population: A cross-sectional study. Noise Heal 13: 64–70 10.4103/1463-1741.73997 21173489

[32] Van Laer L, Carlsson P-I, Ottschytsch N, Bondeson M, Konings A, Vandevelde A, et al (2006) The Contribution of Genes Involved in Potassium- Recycling in the Inner Ear to Noise-Induced Hearing Loss. Hum Mutat 27: 786–795. 16823764 10.1002/humu.20360

[33] Pawelczyk M, Van Laer L, Fransen E, Rajkowska E, Konings A, Carlson P-I, et al (2009) Analysis of 663 gene polymorphisms associated with K ion circulation in the inner ear of patients 664 susceptible and resistant to noise-induced hearing loss. Ann Hum Genet 73: 411–421. 10.1111/j.1469-1809.2009.00521.x 19523148

[34] Zhang W, Zhang Y, Lobler M, Schmitz K, Ahmad A, Pyykko I, et al. (2011) Nuclear entry of hyperbranched polylysine nanoparticles into cochlear cells. Int J Nanomedicine: 535–546. 10.2147/IJN.S16973 PMC3065799 21468356

[35] Lin C-D, Kao M-C, Tsai M-H, Lai C-H, Wei I-H, Tsai M-H, et al (2011) Transient ischemia/hypoxia enhances gentamicin ototoxicity via caspase-dependent cell death pathway. Lab Invest 91: 1092–1106. 10.1038/labinvest.2011.69 21519324

[36] Shin YS, Song SJ, Kang SU, Hwang HS, Choi JW, Lee BH, et al (2012) A novel synthetic compound, 3-amino-3-(4-fluoro-phenyl)-1H-quinoline-2,4-dione, inhibits cisplatin-induced hearing loss by the suppression of reactive oxygen species: In vitro and in vivo study. Neuroscience 232C: 1–12. 10.1016/j.neuroscience.2012.12.008 23246618

[37] Brock PR, Knight KR, Freyer DR, Campbell KCM, Steyger PS, Blakley BW, et al (2012) Platinum-induced ototoxicity in children: a consensus review on mechanisms, predisposition, and protection, including a new International Society of Pediatric Oncology Boston ototoxicity scale. J Clin Oncol 30: 2408–2417. 10.1200/JCO.2011.39.1110 22547603 PMC3675696

[38] Ginisty H, Sicard H, Roger B, Bouvet P (1999) Structure and functions of nucleolin. J Cell Sci 112 6: 761–772. 10036227 10.1242/jcs.112.6.761

[39] Lindenboim L, Blacher E, Borner C, Stein R (2010) Regulation of stress-induced nuclear protein redistribution: a new function of Bax and Bak uncoupled from Bcl-x(L). Cell Death Differ 17: 346–359. 10.1038/cdd.2009.145 19816507

[40] Tajrishi MM, Tuteja R, Tuteja N (2011) Nucleolin: The most abundant multifunctional phosphoprotein of nucleolus. Commun Integr Biol 4: 267–275. 10.4161/cib.4.3.14884 21980556 PMC3187884

[41] Abdelmohsen K, Gorospe M (2012) RNA-binding protein nucleolin in disease. RNA Biol 9: 799–808. 10.4161/rna.19718 22617883 PMC3495746

[42] Jiang B, Zhang B, Liang P, Song J, Deng H, Tu Z, et al (2010) Nucleolin/C23 mediates the antiapoptotic effect of heat shock protein 70 during oxidative stress. FEBS J 277: 642–652. 10.1111/j.1742-4658.2009.07510.x 20050922

[43] Jiang B, Liang P, Wang K, Lv C, Sun L, Tong Z, et al (2014) Nucleolin involved in myocardial ischaemic preconditioning via post-transcriptional control of HSPA1A expression. Cardiovasc Res. 102: 56–67 10.1093/cvr/cvu006 24442868

[44] Daniely Y, Dimitrova DD, Borowiec JA (2002) Stress-dependent nucleolin mobilization mediated by p53-nucleolin complex formation. Mol Cell Biol 22: 6014–6022. 12138209 10.1128/MCB.22.16.6014-6022.2002 PMC133981

[45] Takagi M, Absalon MJ, McLure KG, Kastan MB (2005) Regulation of p53 translation and induction after DNA damage by ribosomal protein L26 and nucleolin. Cell 123: 49–63. 16213212 10.1016/j.cell.2005.07.034

[46] Ishimaru D, Ramalingam S, Sengupta TK, Bandyopadhyay S, Dellis S, Tholanikunnel BG, et al (2009) Regulation of Bcl-2 expression by HuR in HL60 leukemia cells and A431 carcinoma cells. Mol Cancer Res 7: 1354–1366. 10.1158/1541-7786.MCR-08-0476 19671677

[47] Yang M, Tan H, Yang Q, Wang F, Yao H, Wei Q, et al (2006) Association of hsp70 polymorphisms with risk of noise-induced hearing loss in Chinese automobile workers. Cell Stress Chaperones 11: 233–239. 17009596 10.1379/CSC-192R.1 PMC1576471

[48] Chien C-Y, Chang N-C, Tai S-Y, Wang L-F, Wu M-T, Ho K-Y (2012) Heat shock protein 70 gene polymorphisms in sudden sensorineural hearing loss. Audiol Neurootol 17: 381–385. 10.1159/000341815 22922572

[49] Chen J, Guo K, Kastan MB (2012) Interactions of nucleolin and ribosomal protein L26 (RPL26) in translational control of human p53 mRNA. J Biol Chem 287: 16467–16476. 10.1074/jbc.M112.349274 22433872 PMC3351294

[50] Taleb M, Brandon CS, Lee F-S, Harris KC, Dillmann WH, Cunningham LL (2009) Hsp70 inhibits aminoglycoside-induced hearing loss and cochlear hair cell death. Cell Stress Chaperones 14: 427–437. 10.1007/s12192-008-0097-2 19145477 PMC2728278

[51] Cunningham LL, Matsui JI, Warchol ME, Rubel EW (2004) Overexpression of Bcl-2 prevents neomycin-induced hair cell death and caspase-9 activation in the adult mouse utricle in vitro. J Neurobiol 60: 89–100. 15188275 10.1002/neu.20006

[52] Coffin AB, Rubel EW, Raible DW (2013) Bax, Bcl2, and p53 differentially regulate neomycin- and gentamicin-induced hair cell death in the zebrafish lateral line. J Assoc Res Otolaryngol 14: 645–659. 10.1007/s10162-013-0404-1 23821348 PMC3767879

[53] Zhang B, Wang H, Jiang B, Liang P, Liu M, Deng G, et al (2010) Nucleolin/C23 is a negative regulator of hydrogen peroxide-induced apoptosis in HUVECs. Cell Stress Chaperones 15: 249–257. 10.1007/s12192-009-0138-5 19757191 PMC2866999

[54] Willimott S, Wagner SD (2010) Post-transcriptional and post-translational regulation of Bcl2. Biochem Soc Trans 38: 1571–1575. 10.1042/BST0381571 21118128

[55] Wang K, Deng G, Chen G, Liu M, Yi Y, Yang T, et al (2012) Heat shock protein 70 inhibits hydrogen peroxide-induced nucleolar fragmentation via suppressing cleavage and down-regulation of nucleolin. Cell Stress Chaperones 17: 121–130. 10.1007/s12192-011-0292-4 21960124 PMC3227849

[56] Zdebik AA, Wangemann P, Jentsch TJ (2009) Potassium ion movement in the inner ear: insights from genetic disease and mouse models. Physiology (Bethesda) 24: 307–316. 19815857 10.1152/physiol.00018.2009 PMC4415853

[57] Konings A, Van Laer L, Wiktorek-Smagur A, Rajkowska E, Pawelczyk M, Carlsson P-I, et al (2009) Candidate gene association study for noise-induced hearing loss in two independent noise-exposed populations. Ann Hum Genet 73: 215–224. 10.1111/j.1469-1809.2008.00499.x 19183343

[58] Li S, Choi V, Tzounopoulos T (2013) Pathogenic plasticity of Kv7.2/3 channel activity is essential for the induction of tinnitus. Proc Natl Acad Sci U S A 110: 9980–9985. 10.1073/pnas.1302770110 23716673 PMC3683764

[59] Jentsch TJ (2000) Neuronal KCNQ potassium channels: physiology and role in disease. Nat Rev Neurosci 1: 21–30. 11252765 10.1038/35036198

[60] Holt JR, Stauffer EA, Abraham D, Géléoc GSG (2007) Dominant-negative inhibition of M-like potassium conductances in hair cells of the mouse inner ear. J Neurosci 27: 8940–8951. 17699675 10.1523/JNEUROSCI.2085-07.2007 PMC2647843

[61] Zhou X, Wei J, Song M, Francis K, Yu SP (2011) Novel role of KCNQ2/3 channels in regulating neuronal cell viability. Cell Death Differ 18: 493–505. 10.1038/cdd.2010.120 20885443 PMC3017650

[62] Girotto G, Pirastu N, Sorice R, Biino G, Campbell H, D'Adamo A, et al (2011) Hearing function and thresholds: a genome-wide association study in European isolated populations identifies new loci and pathways. J Med Genet 48: 369–374. 10.1136/jmg.2010.088310 21493956

[63] Lu R, Alioua A, Kumar Y, Eghbali M, Stefani E, Toro L (2006) MaxiK channel partners: physiological impact. J Physiol 570: 65–72. 16239267 10.1113/jphysiol.2005.098913 PMC1464300

[64] Manzanares D, Srinivasan M, Salathe ST, Ivonnet P, Baumlin N, Dennis J, et al (2014) IFN-γ reduction of large conductance, Ca2+ activated, voltage-dependent K+ (BK) channel activity in airway epithelial cells leads to mucociliary dysfunction. Am J Physiol Lung Cell Mol Physiol 306: L453–L462. 10.1152/ajplung.00247.2013 24414257 PMC3949055

[65] Hajas A, Szodoray P, Barath S, Sipka S, Rezes S, Zeher M, et al (2009) Sensorineural hearing loss in patients with mixed connective tissue disease: immunological markers and cytokine levels. J Rheumatol 36: 1930–1936. 10.3899/jrheum.081314 19684145

[66] N’Gouemo P (2011) Targeting BK (big potassium) channels in epilepsy. Expert Opin Ther Targets 15: 1283–1295. 10.1517/14728222.2011.620607 21923633 PMC3219529

[67] Cross JH, Arora R, Heckemann R a, Gunny R, Chong K, Carr L, et al (2013) Neurological features of epilepsy, ataxia, sensorineural deafness, tubulopathy syndrome. Dev Med Child Neurol 55: 846–856. 10.1111/dmcn.12171 23924083 PMC4298033

[68] Ignatov A, Lintzel J, Kreienkamp H-J, Schaller HC (2003) Sphingosine-1-phosphate is a high-affinity ligand for the G protein-coupled receptor GPR6 from mouse and induces intracellular Ca2+ release by activating the sphingosine-kinase pathway. Biochem Biophys Res Commun 311: 329–336. 14592418 10.1016/j.bbrc.2003.10.006

[69] Nakayama M, Tabuchi K, Hoshino T, Nakamagoe M, Nishimura B, Hara A (2014) The influence of sphingosine-1-phosphate receptor antagonists on gentamicin-induced hair cell loss of the rat cochlea. Neurosci Lett 561: 91–95. 10.1016/j.neulet.2013.12.063 24397911

[70] Berulava T, Horsthemke B (2010) The obesity-associated SNPs in intron 1 of the FTO gene affect primary transcript levels. Eur J Hum Genet 18: 1054–1056. 10.1038/ejhg.2010.71 20512162 PMC2987405

[71] Guil S, Esteller M (2012) Cis-acting noncoding RNAs: friends and foes. Nat Struct Mol Biol 19: 1068–1075. 10.1038/nsmb.2428 23132386

[72] Skinner MK, Manikkam M, Guerrero-Bosagna C (2010) Epigenetic transgenerational actions of environmental factors in disease etiology. Trends Endocrinol Metab 21: 214–222. 10.1016/j.tem.2009.12.007 20074974 PMC2848884

[73] Mathers JC, Strathdee G, Relton CL (2010) Induction of epigenetic alterations by dietary and other environmental factors 1st ed. Elsevier Inc. 10.1016/B978-0-12-380864-6.00001-8 20933124

[74] Le Prell CG, Dell S, Hensley B, Hall JW, Campbell KCM, Antonelli PJ, et al (2013) Digital music exposure reliably induces temporary threshold shift in normal-hearing human subjects. Ear Hear 33: e44–58. 10.1097/AUD.0b013e31825f9d89 PMC3480981 22885407

[75] Kujawa SG, Liberman MC (2009) Adding insult to injury: cochlear nerve degeneration after “temporary” noise-induced hearing loss. J Neurosci 29: 14077–14085. 10.1523/JNEUROSCI.2845-09.2009 19906956 PMC2812055

[76] Wilhelm M, Zueger T, De Marchi S, Rimoldi SF, Brugger N, Steiner R, et al. (2012) Inflammation and atrial remodeling after a mountain marathon. Scand J Med Sci Sports: 1–7. 10.1111/sms.12030 23253265

[77] Pejovic S, Basta M, Vgontzas AN, Kritikou I, Shaffer ML, Tsaoussoglou M, et al (2013) Effects of recovery sleep after one work week of mild sleep restriction on interleukin-6 and cortisol secretion and daytime sleepiness and performance. Am J Physiol Endocrinol Metab 305: E890–6. 10.1152/ajpendo.00301.2013 23941878 PMC3798707

[78] Lantier L, Fentz J, Mounier R, Leclerc J, Treebak JT, Pehmøller C, et al (2014) AMPK controls exercise endurance, mitochondrial oxidative capacity, and skeletal muscle integrity. FASEB J 28: 3211–3224. 10.1096/fj.14-250449 24652947

[79] Mitchell P, Gopinath B, McMahon CM, Rochtchina E, Wang JJ, Boyages SC, et al (2009) Relationship of Type 2 diabetes to the prevalence, incidence and progression of age-related hearing loss. Diabet Med 26: 483–488. 10.1111/j.1464-5491.2009.02710.x 19646187

[80] Simone MJ, Tan ZS (2011) The role of inflammation in the pathogenesis of delirium and dementia in older adults: a review. CNS Neurosci Ther 17: 506–513. 10.1111/j.1755-5949.2010.00173.x 20553303 PMC6493838

[81] Lin FR, Metter EJ, O’Brien RJ, Resnick SM, Zonderman AB, Ferrucci L (2011) Hearing loss and incident dementia. Arch Neurol 68: 214–220. 10.1001/archneurol.2010.362 21320988 PMC3277836

[82] Koyama A, O’Brien J, Weuve J, Blacker D, Metti AL, Yaffe K (2013) The role of peripheral inflammatory markers in dementia and Alzheimer’s disease: a meta-analysis. J Gerontol A Biol Sci Med Sci 68: 433–440. 10.1093/gerona/gls187 22982688 PMC3693673

[83] Gurgel RK, Ward PD, Schwartz S, Norton MC, Foster NL, Tschanz, JT (2014) Relationship of Hearing Loss and Dementia: A Prospective, Population-Based Study. Otol Neurotol: 1–7. 10.1097/MAO.0000000000000313 PMC4024067 24662628

[84] Fujioka M, Kanzaki S, Okano HJ, Masuda M, Ogawa K, Okano H (2006) Proinflammatory cytokines expression in noise-induced damaged cochlea. J Neurosci Res 83: 575–583. 16429448 10.1002/jnr.20764

[85] Wakabayashi K, Fujioka M, Kanzaki S, Okano HJ, Shibata S, Yamashita D, et al (2010) Blockade of interleukin-6 signaling suppressed cochlear inflammatory response and improved hearing impairment in noise-damaged mice cochlea. Neurosci Res 66: 345–352. 10.1016/j.neures.2009.12.008 20026135

[86] Nash SD, Cruickshanks KJ, Zhan W, Tsai MY, Klein R, Chappell R, et al (2014) Long-term assessment of systemic inflammation and the cumulative incidence of age-related hearing impairment in the epidemiology of hearing loss study. J Gerontol A Biol Sci Med Sci 69: 207–214. 10.1093/gerona/glt075 23739996 PMC4038239

[87] Fujioka M, Okamoto Y, Shinden S, Okano HJ, Okano H, Ogawa K, et al (2014) Pharmacological inhibition of cochlear mitochondrial respiratory chain induces secondary inflammation in the lateral wall: a potential therapeutic target for sensorineural hearing loss. PLoS One 9: e90089 10.1371/journal.pone.0090089 24614528 PMC3948682

[88] Yuan B-C, Su F-M, Wu W-T, Liu W-S, Chiu K-H (2012) A predictive model of the association between gene polymorphism and the risk of noise-induced hearing loss caused by gunfire noise. J Chin Med Assoc 75: 36–39. 10.1016/j.jcma.2011.09.015 22240535

[89] Konings A, Van Laer L, Pawelczyk M, Carlsson P-I, Bondeson M-L, Rajkowska E, et al (2007) Association between variations in CAT and noise-induced hearing loss in two independent noise-exposed populations. Hum Mol Genet 16: 1872–1883. 17567781 10.1093/hmg/ddm135

[90] Lin C-Y, Wu J-L, Shih T-S, Tsai P-J, Sun Y-M, Guo Y-L (2009) Glutathione S-transferase M1, T1, and P1 polymorphisms as susceptibility factors for noise-induced temporary threshold shift. Hear Res 257: 8–15. 10.1016/j.heares.2009.07.008 19643173

[91] Abreu-Silva RS, Rincon D, Horimoto ARVR, Sguillar AP, Ricardo LAC, Kimura L, et al (2011) The search of a genetic basis for noise-induced hearing loss (NIHL). Ann Hum Biol 38: 210–218. 10.3109/03014460.2010.513774 20812880

[92] Konings A, Van Laer L, Michel S, Pawelczyk M, Carlsson P-I, Bondeson M-L, et al (2009) Variations in HSP70 genes associated with noise-induced hearing loss in two independent populations. Eur J Hum Genet 17: 329–335. 10.1038/ejhg.2008.172 18813331 PMC2986160

[93] Chang N-C, Ho C-K, Lin H-Y, Yu M-L, Chien C-Y, Ho K-Y (2011) Association of polymorphisms of heat shock protein 70 with susceptibility to noise-induced hearing loss in the Taiwanese population. Audiol Neurootol 16: 168–174. 10.1159/000317119 20714140

[94] Fortunato G, Marciano E, Zarrilli F, Mazzaccara C, Intrieri M, Calcagno G, et al (2004) Paraoxonase and superoxide dismutase gene polymorphisms and noise-induced hearing loss. Clin Chem 50: 2012–2018. 15345661 10.1373/clinchem.2004.037788

[95] Chang N-C, Ho C-K, Wu M-T, Yu M-L, Ho K-Y (2009) Effect of manganese-superoxide dismutase genetic polymorphisms IVS3-23T/G on noise susceptibility in Taiwan. Am J Otolaryngol 30: 396–400. 10.1016/j.amjoto.2008.08.001 19880028

