# Supplementary figures and images for: Genetic Polymorphisms Associated with Hearing Threshold Shift in Subjects during First Encounter with Occupational Impulse Noise
Source: PLoS One. 2015 Jun 29;10(6):e0130827. doi: 10.1371/journal.pone.0130827 (PMC4488244; doi:10.1371/journal.pone.0130827)

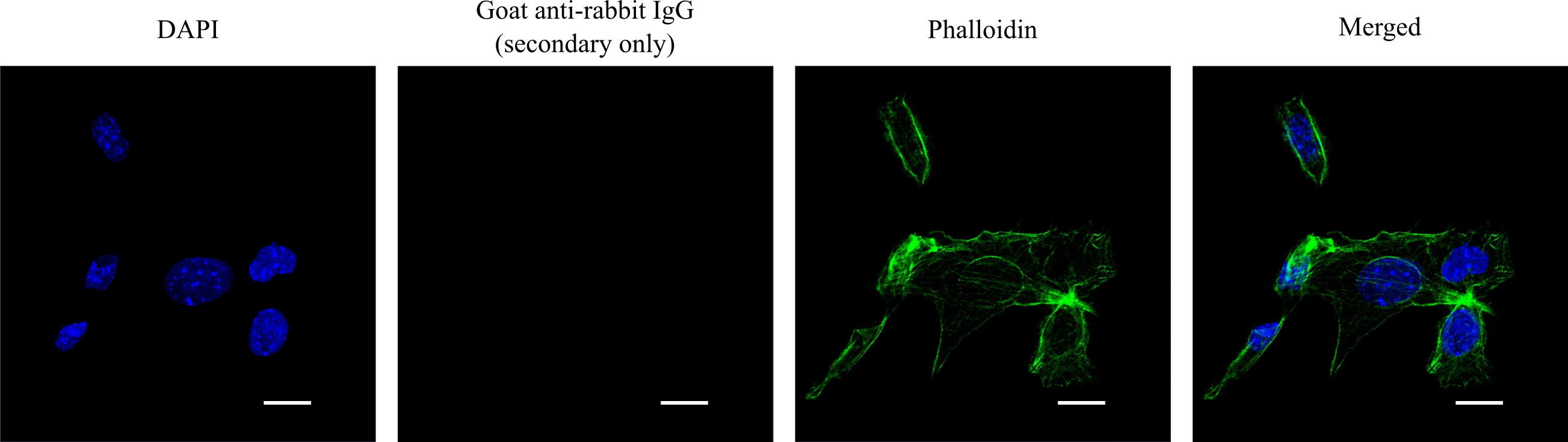

Supplement: S1 Fig — Cultured HEI-OC1 cells with scramble shRNA control immuno-decorated with (B) goat anti-rabbit IgG Texas-Red conjugate used as antibody specific control. Cells were further counterstained with (A) DAPI to show nuclear profile and (C) phalloidin Alexa 488 conjugate to show actin cytoskeletal features. (D) The merge panel clearly shows nuclear and actin profiles and absence of non-specific staining. White scale bar represents 20 μm. (TIF) [file pone.0130827.s001.tif]

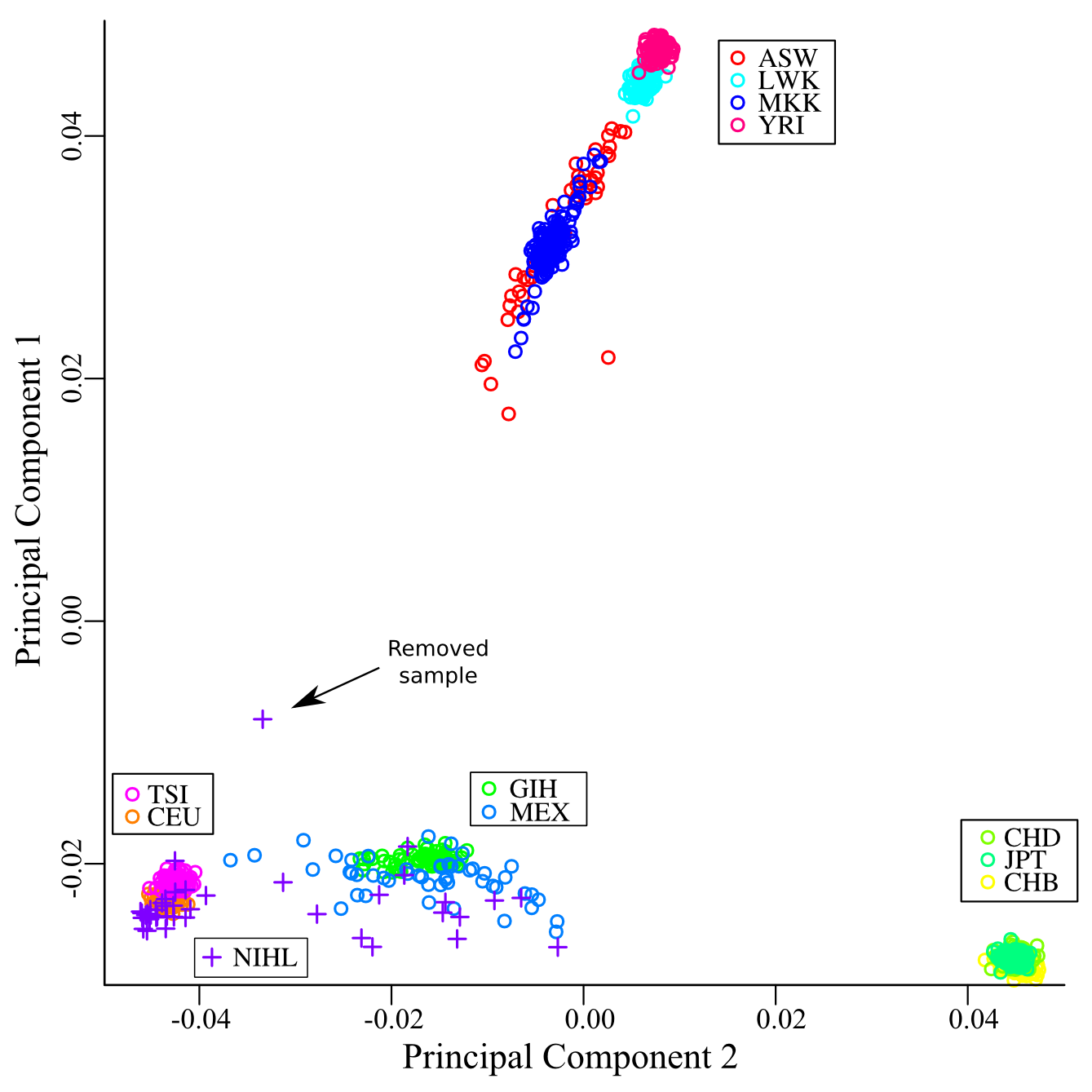

Supplement: S2 Fig — This figure demonstrates the results of the principal component analysis to identify stratification in order to avoid bias. Eleven sub-populations from 1000 Genomes were tested to stratify the NIHL study population. Principal component 1, defined as axis of the linear least square fit of the tested population and principal component 2, which is defined as second axis of the linear least square fit orthogonal to principal component 1, show the largest genetic variance between ASW (African ancestry in Southwest USA), CEU (Utah residents with Northern and Western European ancestry), CHB (Han Chinese in Beijing, China), CHD (Chinese in Metropolitan Denver), GIH (Gujarati Indians in Houston), JPT (Japanese in Tokyo), LWK (Luhya in Webuye, Kenya), MEX (Mexican ancestry in Los Angeles), MKK (Maasai in Kinyawa, Kenya), TSI (Toscans in Italy), YRI (Yoruba in Ibadan). (TIF) [file pone.0130827.s002.tif]
